# Low molecular weight heparin and direct oral anticoagulants influence tumour formation, growth, invasion and vascularisation by separate mechanisms

**DOI:** 10.1038/s41598-019-42738-1

**Published:** 2019-04-18

**Authors:** Sophie Featherby, Yu Pei Xiao, Camille Ettelaie, Leonid L. Nikitenko, John Greenman, Anthony Maraveyas

**Affiliations:** 1 Biomedical Section, University of Hull, Cottingham Road, Hull, HU6 7RX UK; 2 Division of Cancer-Hull York Medical School, University of Hull, Cottingham Road, Hull, HU6 7RX UK

**Keywords:** Tumour angiogenesis, Cell invasion, Drug development

## Abstract

The bidirectional association between coagulation and cancer has been established. However, anticoagulant therapies have been reported to have beneficial outcomes by influencing the vascularisation of the tumours. In this study the influence of a set of anticoagulants on tumour formation, invasion and vascularisation was examined. WM-266-4 melanoma and AsPC-1 pancreatic cancer cell lines were treated with LMWH (Tinzaparin and Dalteparin), and DOAC (Apixaban and Rivaroxaban) and the rate of tumour formation, growth and invasion were measured *in vitro*. In addition, the influence of these anticoagulants on vascularisation was examined using the chorioallantoic membrane assay (CAM) model and compared to the outcome of treatment with Bevacizumab. Using this model the influence of pharmacological concentrations of the anticoagulant on the growth, invasion and vascularisation of tumours derived from WM-266-4 and AsPC-1 cells was also measured *in vivo*. Tinzaparin and Daltepain reduced tumour formation and invasion by the cell lines *in vitro*, but with dissimilar potencies. In addition, treatment of CAM with LMWH reduced the local vascular density beyond that achievable with Bevacizumab, particularly suppressing the formation of larger-diameter blood vessels. In contrast, treatment with DOAC was largely ineffective. Treatment of CAM-implanted tumours with LMWH also reduced tumour vascularisation, while treatment of tumours with Apixaban reduced tumour growth *in vivo*. In conclusion, LMWH and DOAC appear to have anti-cancer properties that are exerted through different mechanisms.

## Introduction

Thrombotic complications are among the leading causes of morbidity and mortality in cancer patients^1,2^ and to date, low molecular weight heparin (LMWH) remains a recommended regime for anticoagulation in cancer patients^2–4^. Favourable biological effects of LMWH in the treatment of cancer patients appear to extend beyond the treatment of thrombosis^5–7^ although these have not definitively translated into clinical gains^8,9^. These beneficial influences include the inhibition of chemotaxis^10,11^, tumour growth^10,12^, metastasis^5,10,13,14^ and the inhibition of angiogenesis^14–19^, and are not mutually exclusive. In contrast, other studies do not point to an anti-proliferative property for LMWH^20,21^. Furthermore, although similarities exist between the different preparation of LMWH^22^, there are differences in physicochemical properties and biological actions of these agents which may explain some of the variability of results in clinical studies^23,24^. LMWH is an anti-coagulant which acts by inhibiting factor Xa (fXa) in an antithrombin-dependant manner. However, preparations of LMWH differ in size and charge^22^ which in turn differentially bind a large variety of proteins within the blood stream^18,25^ resulting in variable responses by the cells. One such outcome of LMWH includes the release of tissue factor pathway inhibitor (TFPI) from endothelial cells^26^. The release of TFPI in turn inhibits the components of the extrinsic pathway of coagulation^26^ as well as constraining angiogenesis by blocking the phosphorylation of VEGF-receptor 2^27^. Furthermore, non-anticoagulant heparins such as S-NACH have been shown to possess anti-oncogenic properties^28^ which further suggests that the beneficial effects of LMWH may be distinct from the anticoagulant function^25^.

The recent development of clinically relevant direct anti-fXa inhibitors provides an opportunity to apportion elements of anti-cancer influence of LMWH to particular mechanisms with greater accuracy. Recently, the use of direct oral anticoagulants (DOAC) have become an option in the treatment of cancer-associated venous thromboembolism^2^. The anticoagulant function of agents such as Apixaban and Rivaroxaban is derived from the inhibition of coagulation factor Xa, and is therefore similar to that of a number of LMWH. However, these agents do not require the function of antithrombin III, which also possesses independent anti-angiogenic properties^29^.

The chorioallantoic membrane (CAM) based model is an established model to explore the anti-angiogenic potential of biological agents and to examine the properties of tumours^30,31^. Recently, a number of studies have used this procedure to examine and compare the influence of preparations of LMWH as anti-angiogenic agents^32–36^. In this study, we have used the CAM model in chick embryo to explore the ability of selected anticoagulants to influence tumour vascularisation, using tumours prepared from MW-266-4 melanoma and AsPC-1 pancreatic cancer cell lines. The CAM were supplemented with the LMWH; Tinzaparin and Dalteparin, and also with direct oral anti-coagulants; Apixaban and Rivaroxaban, and were compared to the effects of the VEGF-receptor blocker; Bevacizumab, *in vivo*. In addition, the direct influence of these anticoagulants on tumour formation, growth and invasion was examined by preparing spheroid tumours which were then implanted into the invasion matrix *in vitro*.

## Material and Methods

### Reagent preparation

Tinzaparin solution (20,000 IU/ml Innohep, LeoPharma Ballerup, Denmark) and Dalteparin solution (25,000 IU/ml Fragmin, Pfizer, Tadworth, UK) were diluted in sterile phosphate buffered saline (PBS) as required. Apixaban and Rivaroxaban were obtained as pure compounds from, Bistrol-Myers Squibb (New York, USA) and Bayer (Leverkusen, Germany) respectively. These were then dissolved gradually in dimethyl sulfoxide (DMSO; 0.1% v/v final concentration) and then diluted to 4 mg/ml in PBS. Controls were also prepared by diluting DMSO. Bevacizumab solution (25 µg/ml Avastin; Roche, Welwyn Garden City, UK) was diluted in sterile PBS as required.

### Cell culture and determination of cell numbers

WM-266-4 melanoma cell line and AsPC-1 pancreatic cancer cell lines (ATCC, Teddington, UK) were cultured in RPMI-1640 supplemented with 10% (v/v) foetal calf serum (FCS). Cell numbers were determined by crystal violet staining as previously described and calculated from a standard curve^37,38^.

### Analysis of tumour spheroid growth and invasiveness *in vitro*

Tumour spheroids were prepared by seeding out WM-266-4 or AsPC-1 cells (2 × 10^4^) in non-adherent Nunclon Sphera 96 wells plates (Thermo Scientific, Warrington, UK) in 50 µl of media. The cells were maintained at 37 °C, 5% CO_2_ for 4 days to permit the formation of tight tumour spheroids. Invasion matrices were prepared by adding 20 µl of Cultrex Spheroid Invasion matrix (Bio-Techne Ltd., Abingdon, UK) in 12-well ibidi µ-chambers (Thistle Scientific, Glasgow, UK) and supplemented with the appropriate media (100 µl) containing a range of concentrations of Tinzaparin or Dalteparin (0–5 IU/ml), Apixaban (0–1 µg/ml) or Rivaroxaban (0–0.6 µg/ml). The tumours were gently transferred to individual invasion assay wells and allowed to set for 1 h at 37 °C. The spheroid tumours were examined daily for up to 72 h by white-light microscopy and photographed using multiple exposures. The multiple images from each sample were then collated and stitched into a composite figure. The observable area of the spheroid and the invasion of the cells into the matrix were analysed using ImageJ software.

### Analysis of tumour formation by measuring tumour intactness *in vitro*

Tumour spheroids were prepared by seeding out WM-266-4 or AsPC-1 cells (2 × 10^4^) in non-adherent Nunclon Sphera 96 wells plates in media (100 µl) containing a range of concentrations of Tinzaparin or Dalteparin (0–5 IU/ml), Apixaban (0–1 µg/ml) or Rivaroxaban (0–0.6 µg/ml). The cells were maintained at 37 °C, 5% CO_2_ for 48 h to permit the formation of the spheroid tumours. The spheroid tumours were examined daily for up to 72 h by white-light microscopy and photographed. The relative tumour intactness was then assessed from the size of the formed tumours as well as the number of detectable tumour particles. However, this assay was not usable as a means of measuring the tumour cell proliferation or tumour spreading.

### Analysis of vascularisation using the chorioallantoic membrane assay model

The study was carried out under the UK home office animal licence 40/3564 and approved by the host institute and all methods were performed in accordance with the relevant guidelines and regulations. The chorioallantoic membrane assay (CAM) were prepared and examined as follows. Fertilized white Leghorn chicken eggs were provided by Henry Stewart & Co. Eggs (Hull, UK) and were stored at 12–18 °C for up to 5 days before use. The eggs were placed in an incubator at 70% relative humidity and 37 °C. On the fifth day, a window of approximately 4 cm^2^ was cut into the shell above the air sack and the shell membrane removed from the exposed area. The window was covered with a plastic cap and the eggs returned to the incubator. On the twelfth day, when the CAM reached around 2 cm across, any CAM test with insufficient vascularisation were excluded. Gelfoam absorbable gelatine pads (Pfizer, Tadworth, UK) were soaked with a range of concentrations of the test reagents or with controls. Test reagents, Tinzaparin (0–5 IU/ml), Dalteparin (0–5 IU/ml) and the VEGF-receptor blocker Bevacizumab (0–12.5 µg/ml), were tested alongside the PBS control. FXa inhibitors Apixaban (0–1 µg/ml) and Rivaroxaban (0–0.6 µg/ml) were examined along with a DMSO-treated control. The pads were then placed on the top of the CAM (Supplemental Fig. 1) and the orientation of the Gelform recorded. The extent of vascularisation within the CAM was examined using a Leica stereomicroscope (Leica/GT Vision, Stansfield, UK) over the following 3 days and Images captured using the Leica Application Suite software (version 2.1.0). The images were obtained from the same positions adjacent to the Gelform to ensure that the same area was examined over the 3 days. The micrographs were grouped and coded with an electronically-generated identification number. The samples were then assigned a vessel density score of 0, if there was a 50% or more reduction in vessel density when compared with reference sets of untreated CAM vessels, or 1 if there was no reduction. The scoring was carried out in 4 fields of view taken from each treatment area. The scores were then averaged for each treatment area on each day and assessed. In order to determine the average diameter of the blood vessels in the samples, the micrographs were generated to include a scale bar was using the Leica Application Suite software and were analysed by ImageJ program.

### Analysis of tumour growth, invasiveness and vascularisation using CAM model, *in vivo*

Tumour were prepared by resuspending WM-266-4 or AsPC-1 cells (2 × 10^6^) within Matrigel matrix (5 µl; BD Bioscience, Wokingham, UK) containing Tinzaparin (5 IU/ml), and Apixaban (0–1 µg/ml) as well as the respective vehicle controls. The Matrigel containing the tumours were permitted to set and then placed on top of the CAM and the orientation of the recorded. The size of the tumour was recorded on each day by white-light microscopy as above. The CAM tests were inspected daily for up to 3 days after which the tumours and surrounding tissue were excised and fixed in 4% (v/v) paraformaldehyde. In order to determine tumour vascularisation, the tumours and the underlying membranes were separated. The underlying membranes were washed three times with PBST (PBS containing 0.5% (v/v) Tween 20), and developed using a FITC-conjugated anti-PECAM-1 antibody (eBioscience/Thermo Scientific, Warrington, UK) diluted 1:400 (v/v) in PBST. The samples were then washed a further three times with shaking (10 min each) and images were acquired using a Ziess Axio Vert.A1 inverted fluorescence microscope with (Carl Zeiss Ltd, Welwyn Garden City, UK). The extent of vascularisation was determined by ImageJ program as above.

### Statistical analysis

Statistical analysis of the data was performed using the Statistical Package for the Social Sciences (SPSS Inc. Chicago, USA) and the calculated means values shown. The number of experiments was as stated in each figure legend ± the calculated standard error of the mean. The significance values were calculated by paired t-test, or the one-way ANOVA (analysis of variance) procedure with Tukey’s honesty significance test.

## Results

### LMWH reduces tumour invasion *in vitro*

**Figure 1:**
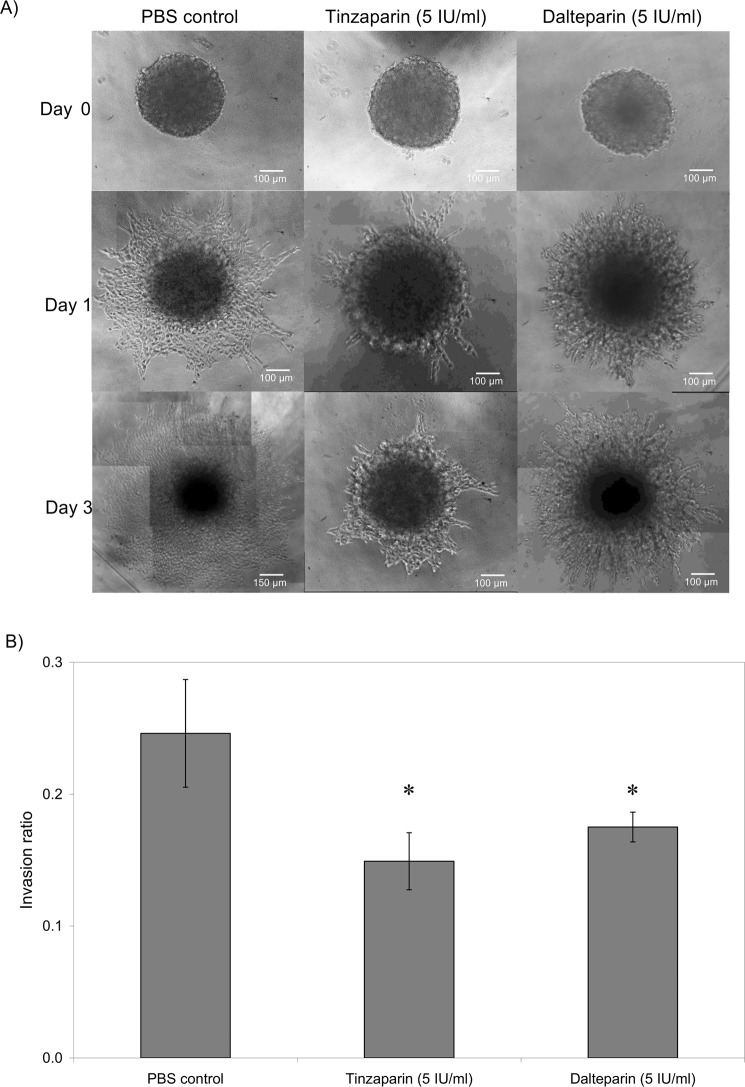
The influence of LMWH on WM-266-4 tumour invasion. Tumour spheroids were prepared by seeding out WM-266-4 (2 × 10^4^) in non-adherent Nunclon Sphera 96 wells plates in 50 µl of media. After 4 days the tumours were transferred to Cultrex Spheroid Invasion matrix in 12-well µ-chambers and supplemented with the appropriate media (100 µl) containing a range of concentrations of Tinzaparin or Dalteparin (0–5 IU/ml). (**A**) The spheroid tumours were examined daily for up to 3 days, by white-light microscopy and photographed. (**B**) The invasion of the cells into the matrix was analysed using ImageJ software. (n = 3, *p < 0.05 vs PBS control).

**Figure 2:**
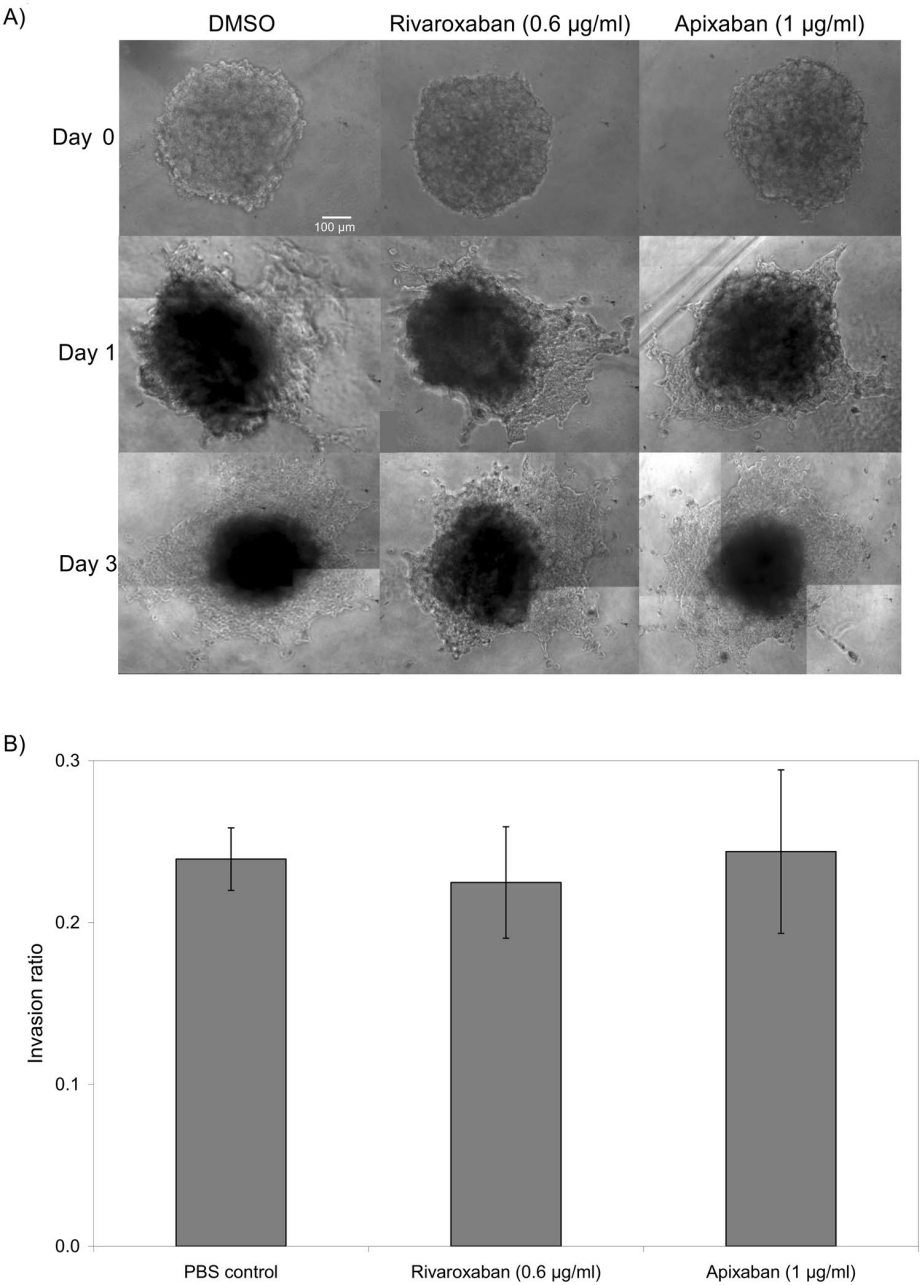
The influence of DOAC on WM-266-4 tumour invasion. Tumour spheroids were prepared by seeding out WM-266-4 (2 × 10^4^) in non-adherent Nunclon Sphera 96 wells plates in 50 µl of media. After 4 days the tumours were transferred to Cultrex Spheroid Invasion matrix in 12-well µ-chambers and supplemented with the appropriate media (100 µl) containing a range of concentrations of Apixaban (0–1 µg/ml) or Rivaroxaban (0–0.6 µg/ml). (**A**) The spheroid tumours were examined daily for up to 3 days, by white-light microscopy and photographed. (**B**) The invasion of the cells into the matrix was analysed using ImageJ software. (n = 3).

Spheroid tumours were prepared by culturing WM-266-4 or AsPC-1 cells in non-adherent plates which were then transferred to the invasion matrix containing the test reagents. The growth and invasion of the tumours was then determined by measuring the size of the main spheroid and the reach of the invading cells. The rate of invasion through the matrix was dissimilar between the two cell lines with WM-266-4 demonstrating a faster rate. Consequently, the data presented depicts the days of measurement most appropriate for each cell line. Incubation of WM-266-4 spheroids with Tinzaparin (5 IU/ml) or Dalteparin (5 IU/ml) significantly reduced tumour invasiveness within the matrix (40% and 30% reduction respectively) by the third day (Fig. 1). However, neither Tinzaparin nor Dalteparin altered tumour invasion by AsPC-1 cells (Not shown). In addition, the inclusion of Rivaroxaban or Apixaban did not have any detectable influence on tumour invasiveness in WM-266-4 (Fig. 2) or AsPC-1 cells (not shown).

### LMWH suppresses tumour intactness/formation

**Figure 3:**
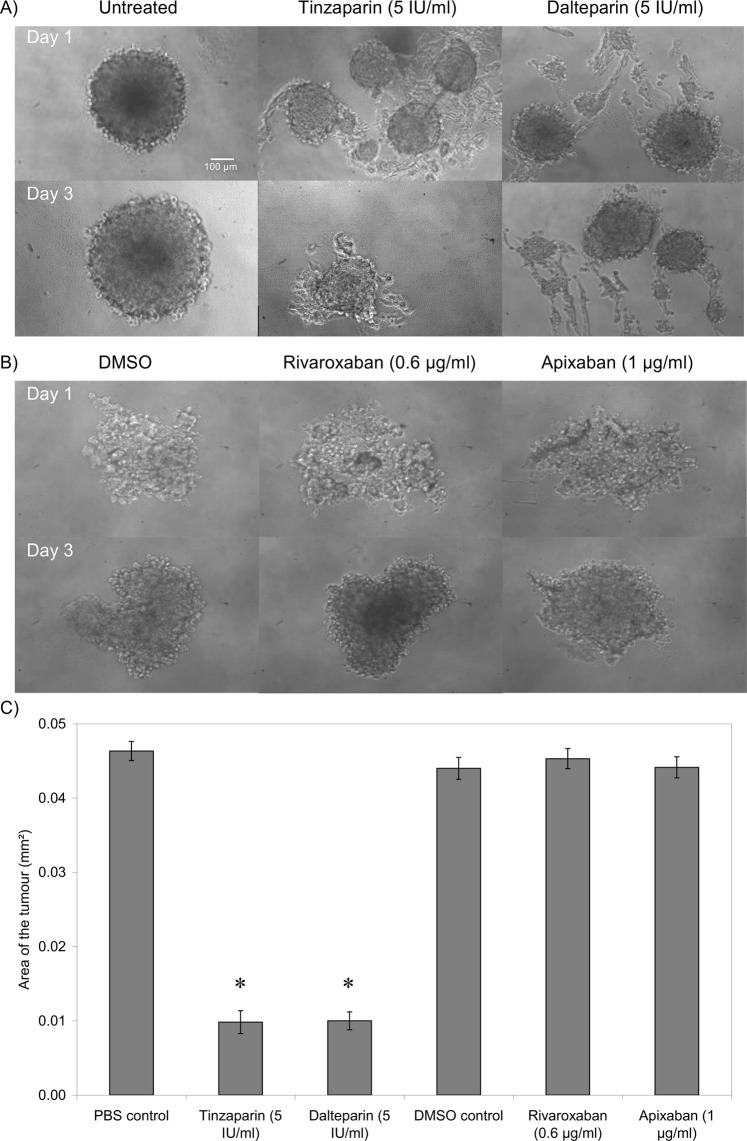
The influence of LMWH and DOAC on tumour formation by WM-266-4 cells. Tumour spheroids were prepared by seeding out WM-266-4 cells (2 × 10^4^) in non-adherent Nunclon Sphera 96 wells plates in media (100 µl) containing a range of concentrations of Tinzaparin or Dalteparin (0–5 IU/ml), Apixaban (0–1 µg/ml) or Rivaroxaban (0–0.6 µg/ml). The cells were maintained at 37 °C, 5% CO_2_ for 48 h to permit the form spheroid tumours. (**A**,**B**) The spheroid tumours were examined daily for up to 72 h, by white-light microscopy and photographed. (**C**) The rate of formation of intact tumours was then assessed from the size of the formed tumours and the number of detectable tumour particles. (n = 3, *p < 0.05 vs PBS control).

**Figure 4:**
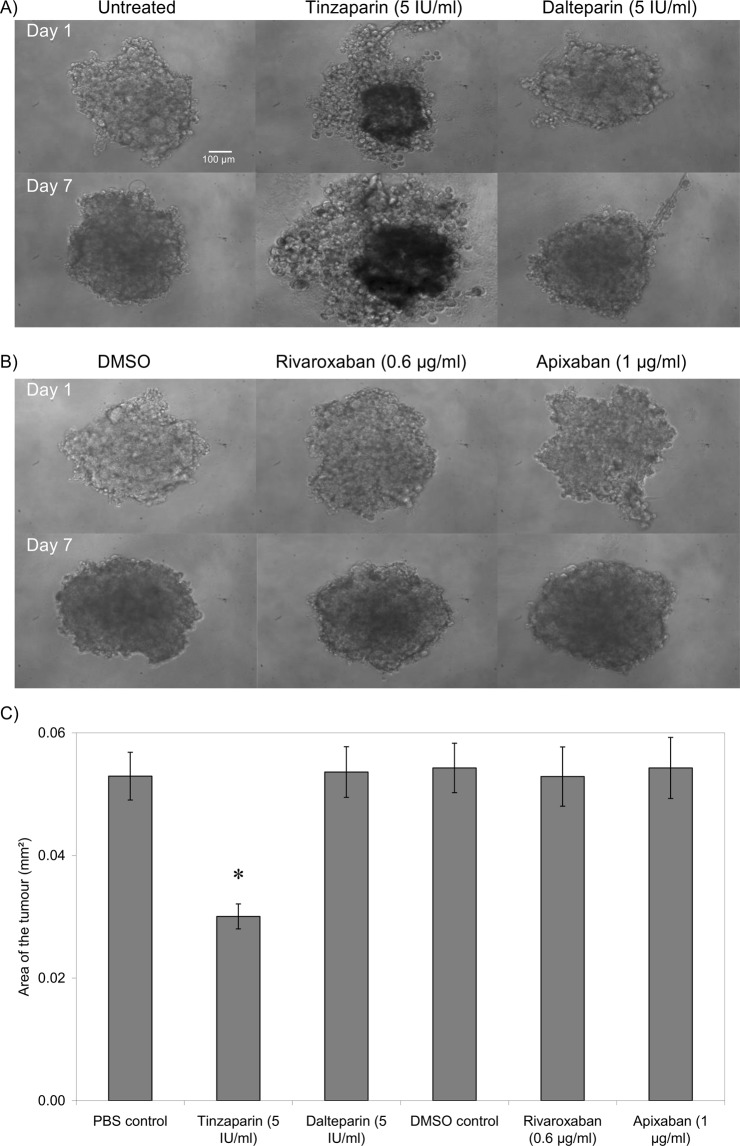
The influence of LMWH and DOAC on tumour formation by AsPC-1 cells. Tumour spheroids were prepared by seeding out AsPC-1 cells (2 × 10^4^) in non-adherent Nunclon Sphera 96 wells plates in media (100 µl) containing a range of concentrations of Tinzaparin or Dalteparin (0–5 IU/ml), Apixaban (0–1 µg/ml) or Rivaroxaban (0–0.6 µg/ml). The cells were maintained at 37 °C, 5% CO_2_ for 48 h to permit the form spheroid tumours. (**A**,**B**) The spheroid tumours were examined daily for up to 72 h, by white-light microscopy. (**C**) The rate of formation of intact tumours was then assessed from the size of the formed tumours and the number of detectable tumour particles. (n = 3, *p < 0.05 vs PBS control).

The formation of spheroid tumours within non-adherent plates was assessed in the presence of the test reagents. In this assay the tumours are formed in non-adherent wells where they cannot spread normally. Consequently, the assay only measures the ability of cells to form tumour mass and does not measure either the tumour-cell proliferation or spreading. Once more, the magnitude of tumour formation was dissimilar between the two cell lines with WM-266-4 forming tighter tumours. Moreover, the inclusion of Tinzaparin markedly reduced tumour intactness in WM-266-4 cells on the first day and significantly inhibited the tumour formation by the third day of experiment (Fig. 3). Supplementation with Dalteparin was less effective although significantly reduced tumour intactness. However, the inclusion of either Rivaroxaban or Apixaban did not significantly alter the tumour formation. The inclusion of Tinzaparin but not Dalteparin also reduced tumour formation in AsPC-1 cells as measured on the seventh day of experiment while the inclusion of either Rivaroxaban or Apixaban was ineffective against this cell line (Fig. 4).

### LMWH reduces vessel density and diameter in a non-fXa dependent manner

**Figure 5:**
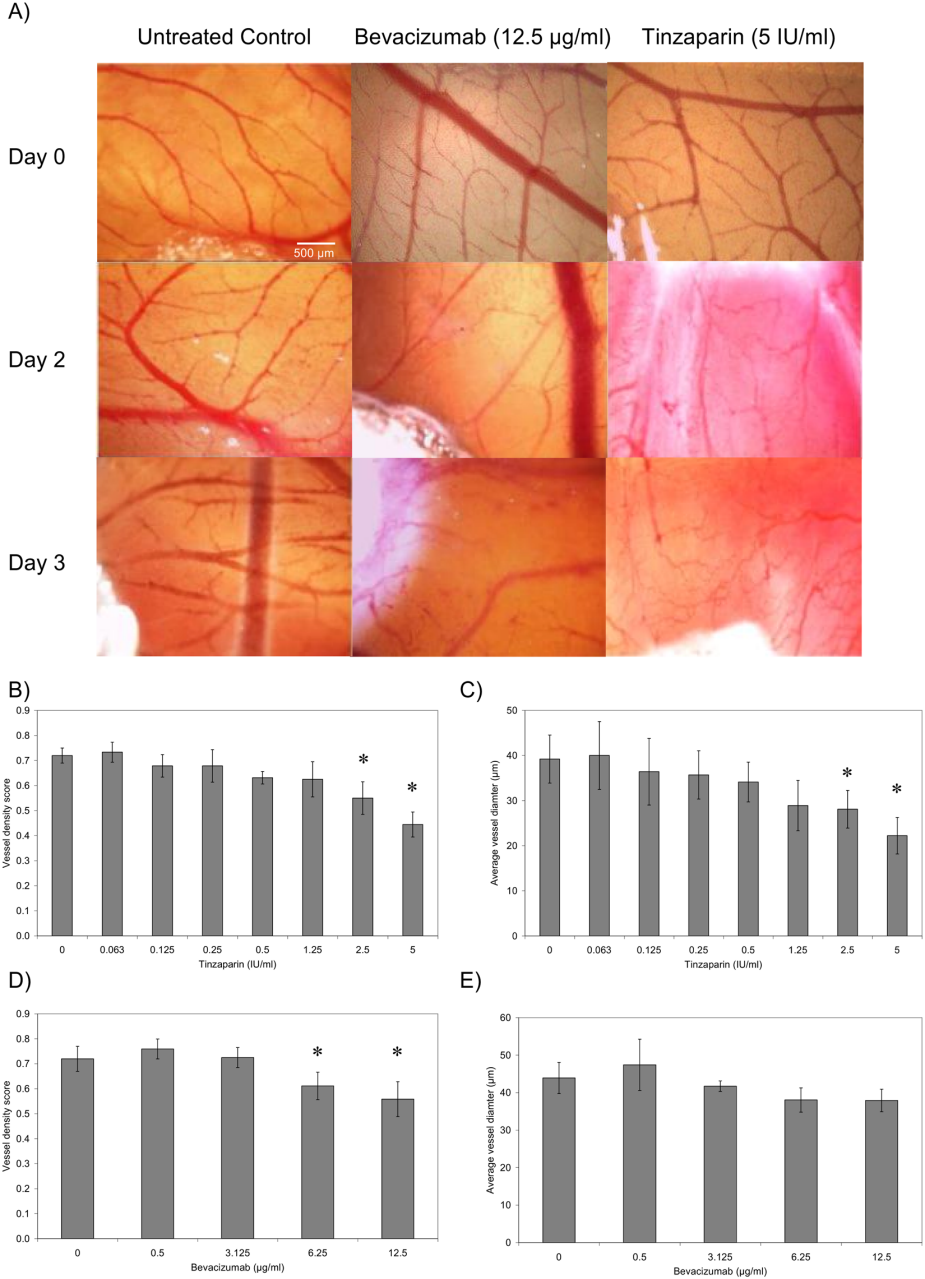
The influence of Tinzaparin and Becacizumab on vascularisation in CAM. The chorioallantoic membrane assay (CAM) were prepared and examined as described in the text. Gelfoam absorbable gelatine pads were soaked with a range of concentrations of Tinzaparin (0–5 IU/ml), Bevacizumab (0–12.5 µg/ml) or PBS control which were placed on the top of the CAM. (**A**) The extent of vascularisation within the CAM was then examined over 3 days and Images captured using Leica Application Suite software. (**B**) The vessel density and (**C**) average vessel diameter was determined in Tinzaparin-treated CAM, and was compared to the (**D**) vessel density and (**E**) average vessel diameter in Bevacizumab-treated samples. (n = 5, *p < 0.05 vs respective PBS control).

**Figure 6:**
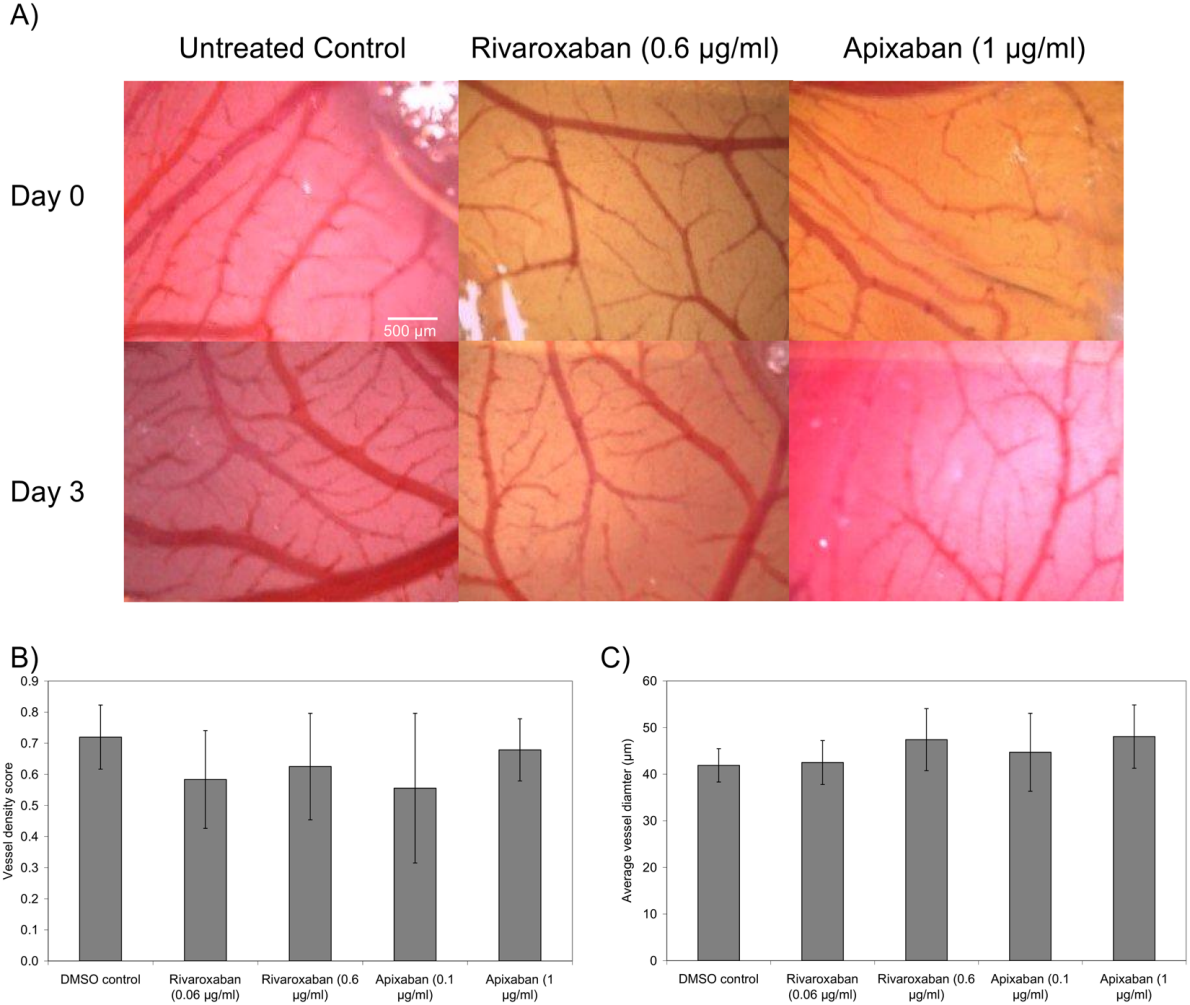
The influence of DOAC on vascularisation in CAM. The chorioallantoic membrane assay (CAM) were prepared and examined as described in the text. Gelfoam pads were soaked with a range of concentrations of Apixaban (0–1 µg/ml) or Rivaroxaban (0–0.6 µg/ml) or DMSO vehicle and placed on the top of the CAM. (**A**) The extent of vascularisation within the CAM was then examined over 3 days and Images captured using Leica Application Suite software. (**B**) The vessel density and (**C**) average vessel diameter was determined and compared to the DMSO-treated samples. (n = 3).

To evaluate the effect of Tinzaparin on CAM vessel formation, Gelfoam pads containing Tinzaparin (0–5 IU/ml) were placed on the CAM for 3 days and recorded daily (Fig. 5A). Images were obtained from the same area with reference to the position of the Gelform. Tinzaparin produced a dose-dependent decrease in vessel density score and vessel diameter with the highest dose used (5 IU/ml) causing 43% inhibition of total vessel density, compared to PBS control (Fig. 5B). This was concurrent with a 46% reduction in average vessel diameter in the treated samples compared to the control (Fig. 5C). For comparison, the influence of the VEGF blocker, Bevacizumab was examined alongside. The inclusion of Bevacizumab within the Gelform pads reduced CAM vessel density score producing 14% inhibition with 12.5 µg/ml (Fig. 5D) without significantly affecting the average diameter of the vessels formed (Fig. 5E). In contrast, inclusion of the either of the two direct fXa inhibitors Rivaroxaban or Apixaban did not alter the vessel density (Fig. 6A,B) or average diameter (Fig. 6A,C).

### LMWH reduces tumour-induced vascularisation

**Figure 7:**
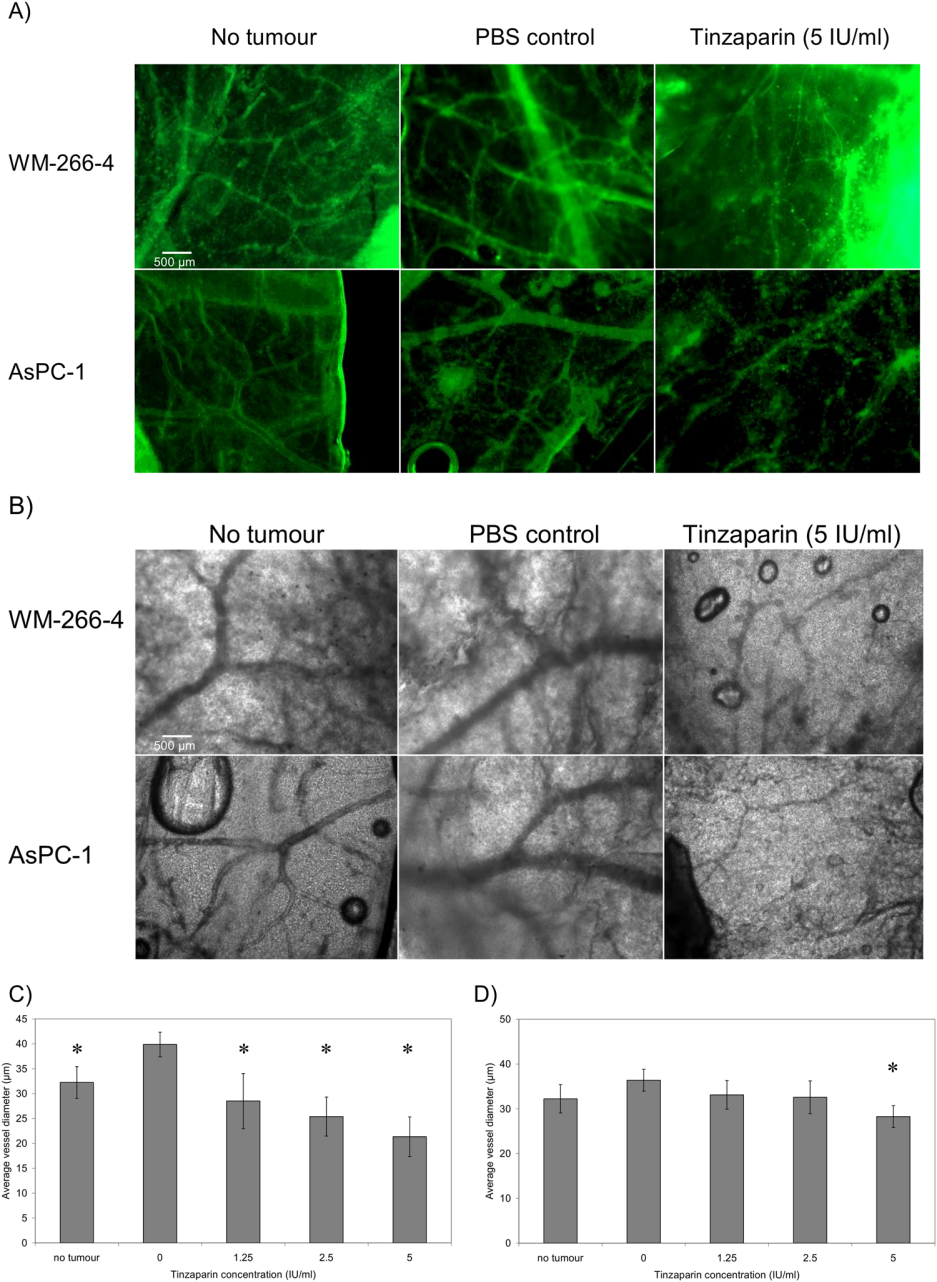
The influence of Tinzaparin on vascularisation of tumour implants. Tumour were prepared by resuspending WM-266-4 or AsPC-1 cells (2 × 10^6^) within Matrigel matrix (5 µl), placed on top of the CAM and permitted to set. The test samples were supplemented with Tinzaparin (5 IU/ml) or PBS controls. The tumours and surrounding tissue were then excised and fixed in 4% (v/v) paraformaldehyde. The tumours were then removed and the underlying membrane probed with FITC-conjugated anti-PECAM-1 antibody and observed by (**A**) fluorescence as well as by (**B**) white-light microscopy. The extend of vascularisation beneath the tumour was determined by imaging and the average vessel diameter around the WM-266-4 (**C**) and AsPC-1 (**D**) tumours was determined. (n = 4, *p < 0.05 vs respective PBS control).

**Figure 8:**
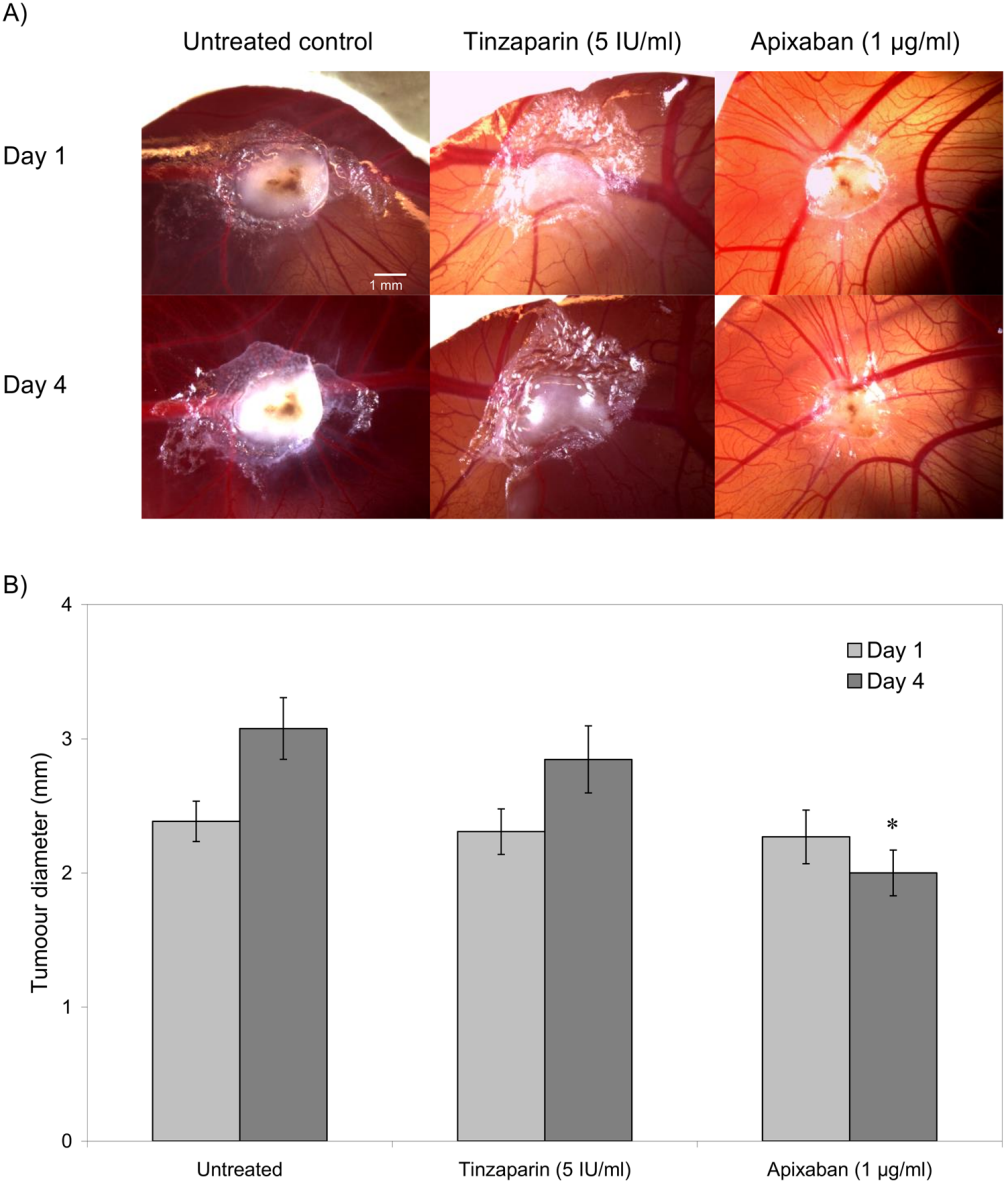
The influence of Tinzaparin and Apixaban on growth of tumour implants. Tumour were prepared by resuspending WM-266-4 cells (2 × 10^6^) within Matrigel matrix (5 µl), placed on top of the CAM and permitted to set. The test samples were supplemented with Tinzaparin (5 IU/ml) or Apixaban (1 µg/ml) as well as untreated controls. (**A**) The tumours were observed over period of 4 days and the size of the of the tumours was determined. (n = 4, *p < 0.05 vs the untreated sample on the respective day).

In addition to examining the direct influence of the above anticoagulants on vascularisation, the outcome on the tumour growth and vascularisation was evaluated, *in vivo*. WM-266-4 and AsPC-1 tumours were implanted on the CAM and the tumours were permitted to grow and vascularise over 3 days. The tumours were then excised and the underlying membrane was probed using an FITC-conjugated rabbit polyclonal anti-PECAM-1 antibody. The membranes were examined by fluorescence (Fig. 7A) and white-light (Fig. 7B) microscopy. Implantation of the tumours spheroids (embedded within Matrigel), derived from either WM-266-4 or AsPC-1 cells resulted in increased vessel density and average vessel diameter at the locality of the tumour. Moreover, supplementation of the WM-266-4 and AsPC-1 cells with Tinzaparin (5 IU/ml) reduced the vascularisation, particularly interfering with the formation of larger vessels (Fig. 7C,D). Treatment with Tinzaparin (5 IU/ml) did not affect the rate of WM-266-4 tumour growth while in contrast, supplementation with Apixaban (1 µg/ml) partially reduced the growth of the implanted tumours compared to the untreated sample (Fig. 8).

## Discussion

It has previously been shown that treatment of cancer patients with LMWH has beneficial influences that extend beyond the point of withdrawal^39^ and in addition to the anti-coagulant activity. These additional influences appear to include the ability of LMWH to interfere with mechanisms including cellular signalling leading to tumour growth and angiogenesis^5,7,10,19,23^. This study aimed to examine the direct effects of Tinzaparin and Dalteparin on tumour cells, and to compare the outcomes to those by Rivaroxaban and Apixaban. All these anticoagulants function by inhibiting the coagulation factor Xa. However, the outcome of inclusion of LMWH were different to those of DOAC. Therefore, it appears that these agents may have functions beyond the inhibition of fXa. The ability of LMWH to alter the behaviour of cancer cells has been demonstrated previously^5^. We previously reported that Dalteparin was capable of reducing the expression of tissue factor through mechanisms that involved the suppression of NFκB activity, by blocking growth factor receptors^40^. In addition, treatment of cell lines also suppressed the invasive and migratory properties of the cells^41,42^. The activation of NFκB by growth factor receptors has been well documented and the blocking of various growth factor receptors by LMWH demonstrated^40^. In addition, heparins have been shown to lower tumour cell adhesion^10^. This action appears to affect different adhesion receptors and includes cell-cell interaction through selectins^5,13^ and adhesion to endothelial cells via ICAM^43–45^. Furthermore, this property of LMWH is not dependent on the anti-coagulant function^46^. In our study, both Tinzaparin and Dalteparin were capable of preventing tumour spheroid formation but in a cell type dependent manner. Furthermore, this ability was dissimilar in the two agents tested, with Tinzaparin exhibiting a greater potency. However, at present we cannot comment on the benefits, or alternatively detrimental outcomes of this property of Tinzaparin.

The anti-angiogenic influence of Tinzaparin was also compared to that of Bevacizumab and the outcome on vessel density and diameter was measured. Tinzaparin was able to reduce the vessel density, particularly the number of larger diameter vessels, in the treated CAM. Furthermore, since the implantation of the tumours increased the density of the underlying vessels, this reduction was more tangible. In contrast, incubation with either DOAC had no detectable influence on vascularistaion. These observation suggest that the anti-angiogenic property of Tinzaparin are also independent of its anti-fXa action. In comparison, Bevacizumab was able to reduce vessel density but did not alter the average vessel diameter. Bevacizumab acts through blocking VEGF receptor and impedes the formation of the capillaries and smaller vessels. However, LMWH seems to interfere with the cellular binding of a number of growth factors. These include the receptors for VEGF, bFGF, EGF^40^ and PDGF^18^ which are fundamental in the formation of larger blood vessels. For example, the ability of LMWH to block PDGF^18^, can alter the pericyte recruitment and maturation of vessel walls. Consequently, Tinzaparin is capable of influencing both the endothelial cells and surrounding pericytes. In contrast, Bevacizumab only hampers the formation of capillaries which primarily consist of endothelial cells.

Finally, the examination of either the CAM-implanted tumours, or the tumour growth *in vitro* did not show any differences in tumour growth on treatment with either LMWH. However, some reduction in the size of the tumour was detected following treatment of cells with Apixaban. This novel reduction in tumour size was attributed to the lower rate of cell proliferation (Featherby *et al*., unpublished data) but the mechanism was not investigated further, in this study.

The anticoagulant function of LMWH arises from its ability to provide a surface for the approximation of the coagulation factor Xa (fXa) and antithrombin III. However, LMWH is also capable of interfering with other mechanisms due to the ability to interact with a large range of proteins within the bloodstream and on the surface of the cells. Among these are a number of receptors involved in cell adhesion, cell migration and signals that control vascularisation and angiogenesis. In conclusion, this study has highlighted the dissimilar outcomes of treatment of cancer cells with therapeutic concentrations of Tinzaparin, Dalteparin, Apixaban and Rivaroxaban on tumour growth, invasion and vascularisation. We have established some of the non-anticoagulant functions of LMWH and elucidated the mode action of these therapeutic agents.

## Supplementary information

Supplementary information
