# Supplementary material for: Low molecular weight heparin and direct oral anticoagulants influence tumour formation, growth, invasion and vascularisation by separate mechanisms
Source: Sci Rep. 2019 Apr 18;9:6272. doi: 10.1038/s41598-019-42738-1 (PMC6472388; doi:10.1038/s41598-019-42738-1)

**Low molecular weight heparin and direct oral anticoagulants influence tumour formation, growth, invasion and vascularisation by separate mechanisms**

Sophie Featherby, Yu Pei Xiao, Camille Ettelaie, Leonid Nikitenko, John Greenman, Anthony Maraveyas

Supplemental Figure 1


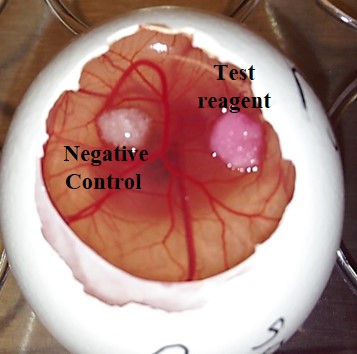

Supplement: Supplementary file 1 — Supplementary information [file 41598_2019_42738_MOESM1_ESM.doc]
